# Good Syndrome in a Young Woman: An Unusual Presentation

**DOI:** 10.7759/cureus.52705

**Published:** 2024-01-22

**Authors:** Sandra D Rebelo, Tiago Ferreira, Teresa Pacheco, Susana L Silva, Ana Tornada

**Affiliations:** 1 Serviço de Medicina Interna, Hospital de Santa Maria - Centro Hospitalar Universitário Lisboa Norte, Lisbon, PRT; 2 Serviço de Oncologia Médica, Hospital de Santa Maria - Centro Hospitalar Universitário Lisboa Norte, Lisbon, PRT; 3 Serviço de Oncologia Médica, Faculdade de Medicina da Universidade de Lisboa, Lisbon, PRT; 4 Serviço de Oncologia Médica, Instituto de Medicina Molecular João Lobo Antunes, Lisbon, PRT; 5 Serviço de Imunoalergologia, Hospital de Santa Maria - Centro Hospitalar Universitário Lisboa Norte, Lisbon, PRT; 6 Serviço de Imunoalergologia, Faculdade de Medicina da Universidade de Lisboa, Lisbon, PRT; 7 Serviço de Imunoalergologia, Instituto de Medicina Molecular João Lobo Antunes, Lisbon, PRT; 8 Serviço de Medicina Interna, Faculdade de Medicina da Universidade de Lisboa, Lisbon, PRT

**Keywords:** recurrent infections, hypogammaglobulinemia, immunodeficiency, thymoma, good syndrome

## Abstract

Good Syndrome is a rare disease that comprises the presence of a thymoma, immunodeficiency, and recurrent opportunistic infections.

We report the case of a young woman who was diagnosed with Good Syndrome, who had a long-term history of recurrent infections, often due to atypical agents, and who also had a previous history of immunodeficiency and a B1 thymoma invading the large vessels, lung, and pericardium (Masaoka stage IV). She underwent surgical resection of the mediastinal mass, requiring vena cava superior reconstruction due to the extent of invasion, followed by adjuvant radiotherapy and immunoglobulin G supplementation. Despite relative stability in the subsequent years, without serious infections, after three years she had a thymoma recurrence requiring a new therapeutic approach.

This case highlights the importance of a thorough investigation of the underlying causes of recurrent infections, which may be the result of an immunodeficiency secondary to malignancy. In young patients, early diagnosis is crucial to avoid disease progression and to reduce mortality rates. To achieve such outcomes, a multidisciplinary team and a comprehensive therapeutic strategy are necessary.

## Introduction

Good Syndrome (GS) is a rare disease first described by Good et al in 1954, characterised by the association of hypogammaglobulinemia (immunodeficiency) and thymoma, which usually affects adults between 40 and 70 years old [1]. 

Patients with GS feature absent or significantly reduced B cell counts, a decreased count or functional impairment of T cells, and reversal of CD4+/CD8+ ratio, predisposing affected individuals to recurrent/severe infections [2]. Although respiratory tract infections are the most common, usually due to encapsulated organisms, other infections may occur due to opportunistic agents [1]. GS may also be associated with autoimmune and haematological manifestations [2].

In the presence of recurrent infections, it is mandatory to exclude underlying primary/secondary immunodeficiency and its aetiology. In particular, when a mediastinal mass is present, distinguishing between lymphoproliferative disorders and thymoma is crucial to the diagnosis of GS.

## Case presentation

We report the case of a 38-year-old woman, admitted in October 2018, for pyelonephritis and diarrhoea. There was a history of recurrent urinary tract infections without any microorganism isolation, leading to multiple courses of antibiotics along with involuntary weight loss (10% of her body mass index) over the previous 6 months. She had no relevant family or personal medical history.

**Figure 1:**
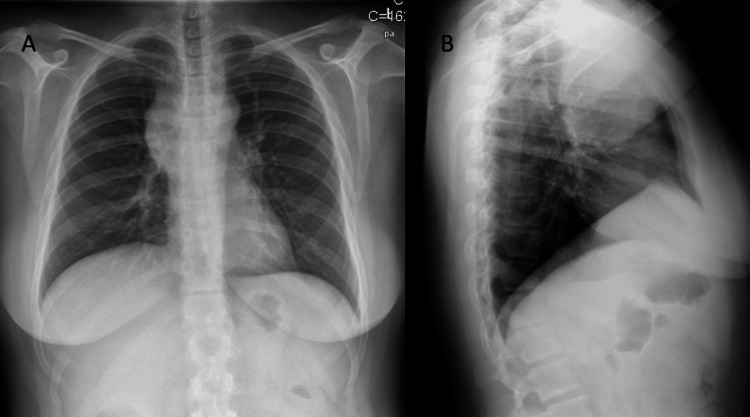
Posteroanterior (A) and lateral (B) thorax radiography showing enlargement of the mediastinum (lobulated mass of 9.0 cm).

**Figure 2:**
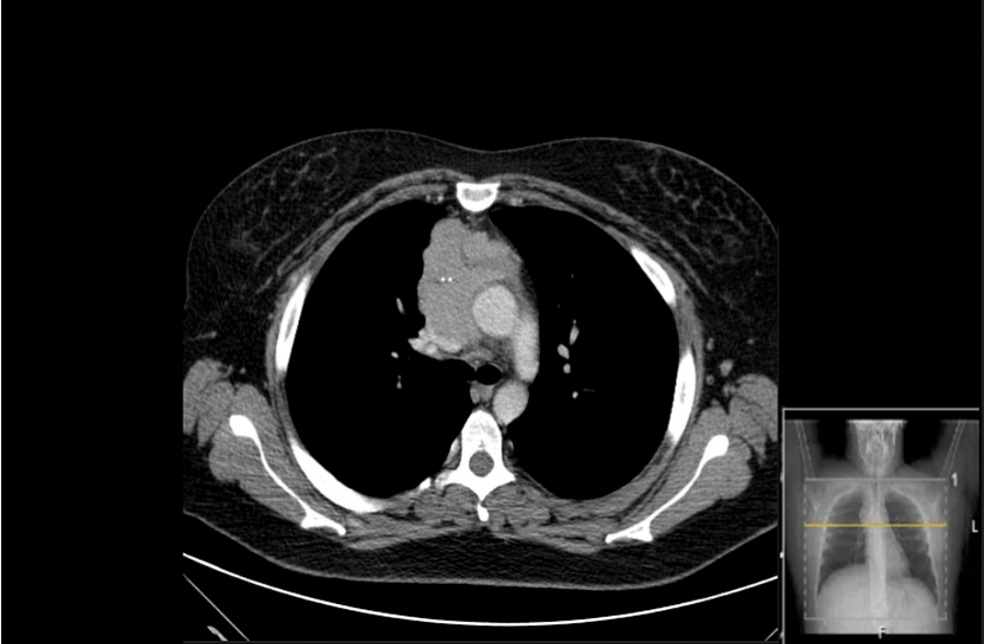
CT-Chest showing a heterogeneous anterior mediastinal solid lesion (7.9x6.7x6.2cm), with areas of central hypocaptation and millimetre calcification, along with lobulated contours, resulting in a significant reduction in the caliber of the distal strand of the left brachiocephalic vein, superior vena cava and contact with the right atrial appendage.

During admission, no microorganism was detected in multiple urine cultures, including bacteria, parasites, mycobacteria, or yeast. Notably, stool culture revealed an opportunistic coinfection of *Clostridium difficile* and *Campylobacter jejuni*. Further investigation revealed hypogammaglobulinemia, with decreased serum IgA and IgM. Extended immunophenotyping of peripheral blood lymphocytes showed low B and T cell counts, along with an inverted CD4/CD8 ratio, likely due to CD8+ T cell expansion. There was no evidence of monoclonal gammopathy or complement consumption. Thoracic radiography and computed tomography scan (Figure 1 and Figure 2, respectively) revealed an anterior mediastinal solid mass.

**Figure 3:**
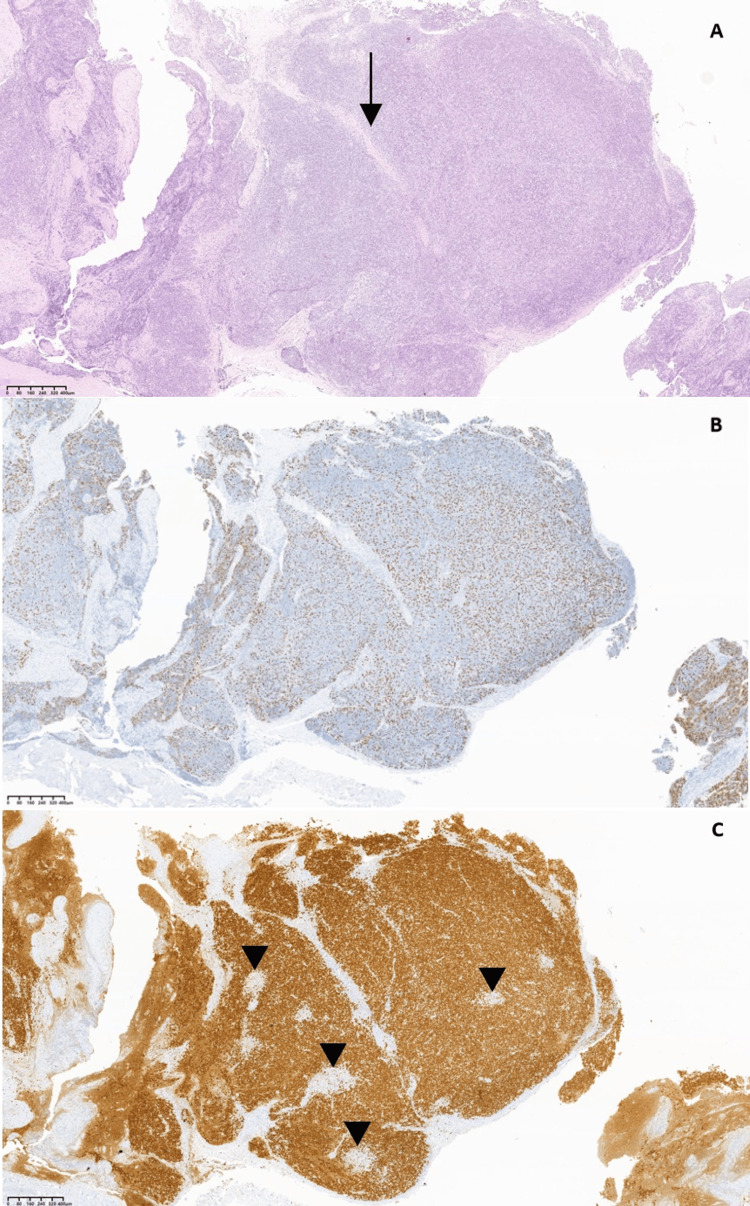
Type B1 thymoma. (A) Encapsulated tumor with fibrous bands (arrow) separating it into lobules, composed of scattered epithelial cells, positive in p40 (B) in a dense background of immature T cells, TdT+ (C); foci of medullary differentiation (arrowhead) are present. Photos courtesy of Dr. Mafalda Pinho Fialho and Dr. Cristina Ferreira (Hospital de Santa Maria, Centro Hospitalar Universitário Lisboa Norte, Lisbon, Portugal).

**Figure 4:**
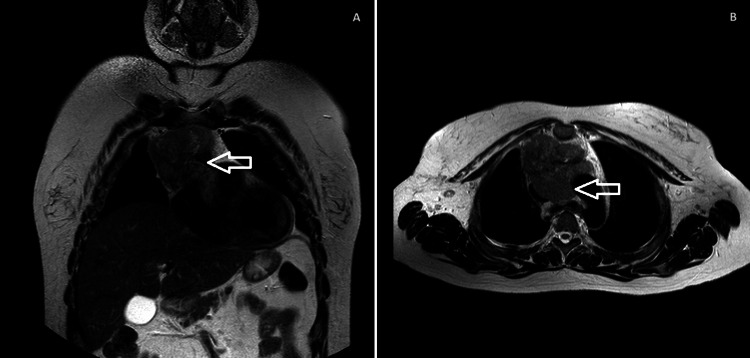
Thoracic magnetic resonance imaging shows coronal (A) and axial (B) views of an anterior mediastinum mass (thymoma) with invasion of the left brachycephalic venous trunk and pericardium. The superior vena cava and ascending aorta are in continuity with the lesion, resulting in significant compression of the superior vena cava.

The histology biopsy (Figure 3), obtained through video-assisted thoracoscopic surgery, confirmed type B1 thymoma, and magnetic resonance imaging (Figure 4) confirmed invasion of adjacent lung and pericardium (Masaoka stage IV) [3].

The mass was surgically resected with positive margins, requiring superior vena cava reconstruction followed by adjuvant radiotherapy. Three years later, follow-up revealed thymoma recurrence with lung and pleural invasion. Six cycles of first-line chemotherapy (cyclophosphamide, doxorubicin, and cisplatin) were performed, followed by pulmonary metastasis resection. A few months later, pleural recurrence was again identified and chemotherapy was resumed, with cisplatin, with stabilisation of the disease.

Subsequent to the diagnosis, the patient has received monthly intravenous immunoglobulin G replacement therapy. Although she continued to experience some mild infections, even of atypical agents such as *Ureaplasma urealyticum*, she did not require additional hospitalizations.

## Discussion

This case is noteworthy due to the patient's young age at presentation and rapid progression of symptoms. The absence of protocols for diagnosis and management makes its approach particularly challenging [1,4]. In addition, this case outlines the importance of considering an underlying immunodeficiency in young adults presenting with recurrent infections, especially due to infrequent agents or requiring admission [1,4]. 

The etiopathogenesis of GS remains unclear [2]. In most cases, the diagnosis of thymoma precedes hypogammaglobulinemia, although the pathophysiological link between thymoma and autoimmunity/immunodeficiency is uncertain and warrants further investigation [4].

Several treatment options have been proposed, but none has demonstrated a significant impact on patient prognosis. Complete thymectomy stands out as the most important determinant of a favourable prognosis, preventing local progression, but it is usually not associated with changes in the immunological status [1-3]. Continuous monitoring of these patients is crucial, even though B1 thymomas are considered low risk, with an overall survival rate of approximately 90% over 20 years [3]. For patients with Masaoka stage III or IV thymomas, the 5-year survival rates range from 93% to 64% for patients submitted to total/subtotal resection respectively [3,4]. Treatment options for recurrent thymoma include surgery, radiation therapy, and chemotherapy agents, such as etoposide, pemetrexed, or paclitaxel [3,5].

In addition, coexisting immunodeficiency significantly increases mortality, primarily due to severe infections [4]. The administration of Immunoglobulin G is essential in reducing infections and hospitalizations and improving patients' quality of life [4].

Autoimmune diseases may also complicate GS, requiring immunosuppressive treatments, despite their limited benefits and poor outcomes. Pure red cell aplasia and myasthenia gravis are the most common, with an incidence of 16% and 35%, respectively. Additionally, other associated disorders include lichen planus, lupus erythematosus, polymyositis, rheumatoid arthritis, thyroiditis, Sjogren’s syndrome, intestinal inflammatory disease, and haematologic manifestations such as aplastic and hemolytic anaemia. A clearer understanding of the pathogenesis underlying immunodeficiency in Good Syndrome may lead to more focused therapies and promote the development of novel drug targets for patients with antibody-mediated autoimmune disorders [4].

## Conclusions

In conclusion, this case stands out not for bacterial or opportunistic infections but rather due to the oncological condition, considering the type, localization, and severity of recurrence, all linked to high mortality. In addition, the survival rates can also be adversely affected by the age of the patients and the presence of concurrent autoimmune and malignant diseases. These conditions highlight the importance of a close and prolonged follow-up.

A multidisciplinary team and a comprehensive therapeutic approach are crucial to improving prognosis.
